# Plant Stomata: An Unrealized Possibility in Plant Defense against Invading Pathogens and Stress Tolerance

**DOI:** 10.3390/plants12193380

**Published:** 2023-09-25

**Authors:** Sandipan Meddya, Shweta Meshram, Deepranjan Sarkar, Rakesh S, Rahul Datta, Sachidanand Singh, Gosangi Avinash, Arun Kumar Kondeti, Ajit Kumar Savani, Thiyagarajan Thulasinathan

**Affiliations:** 1 School of Agriculture, Lovely Professional University, Phagwara 144411, India; 2 Department of Agriculture, Integral Institute of Agricultural Science and Technology, Integral University, Lucknow 226026, India; deep.gogreen@gmail.com; 3 Department of Soil Science and Agricultural Chemistry, Uttar Banga Krishi Viswavidyalaya, Pundibari, Cooch Behar 736165, India; rakisavan.940@gmail.com; 4 Department of Geology and Pedology, Faculty of Forestry and Wood Technology, Mendel University in Brno, 61300 Brno, Czech Republic; rahulmedcure@gmail.com; 5 Department of Biotechnology, Smt. S. S. Patel Nootan Science and Commerce College, Sankalchand Patel University, Visnagar 384315, India; drsachinbioinfo@gmail.com; 6 Department of Biochemistry, Punjab Agricultural University, Ludhiana 141027, India; avinashgosangi@gmail.com; 7 Department of Agronomy, Acharya N.G. Ranga Agricultural University, Regional Agricultural Research Station, Nandyal 518502, India; k.arunkumar@angrau.ac.in; 8 Department of Plant Pathology, Assam Agricultural University, Jorhat 785013, India; ajitkumaragrico@gmail.com; 9 Department of Plant Biotechnology, Centre for Plant Molecular Biology and Biotechnology, Tamil Nadu Agricultural University, Coimbatore 641003, India; stthiyaga@gmail.com

**Keywords:** biotic and abiotic stresses, stomatal responses, defense mechanisms, signaling components, cytosolic Ca^2+^, abscisic acid

## Abstract

Stomata are crucial structures in plants that play a primary role in the infection process during a pathogen’s attack, as they act as points of access for invading pathogens to enter host tissues. Recent evidence has revealed that stomata are integral to the plant defense system and can actively impede invading pathogens by triggering plant defense responses. Stomata interact with diverse pathogen virulence factors, granting them the capacity to influence plant susceptibility and resistance. Moreover, recent studies focusing on the environmental and microbial regulation of stomatal closure and opening have shed light on the epidemiology of bacterial diseases in plants. Bacteria and fungi can induce stomatal closure using pathogen-associated molecular patterns (PAMPs), effectively preventing entry through these openings and positioning stomata as a critical component of the plant’s innate immune system; however, despite this defense mechanism, some microorganisms have evolved strategies to overcome stomatal protection. Interestingly, recent research supports the hypothesis that stomatal closure caused by PAMPs may function as a more robust barrier against pathogen infection than previously believed. On the other hand, plant stomatal closure is also regulated by factors such as abscisic acid and Ca^2+^-permeable channels, which will also be discussed in this review. Therefore, this review aims to discuss various roles of stomata during biotic and abiotic stress, such as insects and water stress, and with specific context to pathogens and their strategies for evading stomatal defense, subverting plant resistance, and overcoming challenges faced by infectious propagules. These pathogens must navigate specific plant tissues and counteract various constitutive and inducible resistance mechanisms, making the role of stomata in plant defense an essential area of study.

## 1. Introduction

Stomata, microscopic pores on the surface of leaves, enable plants to exchange gases with their surroundings, facilitating water evaporation through transpiration and the uptake of carbon dioxide (CO_2_) for photosynthesis. Additionally, stomata serve as essential entry points for phytopathogen endophytic colonization due to their connection between internal plant tissues and the external environment. As a result, plants have developed the ability to adjust their stomatal apertures in response to pathogens, hormones, and various environmental factors, including abscisic acid, light, air humidity, and CO_2_ [1]. Despite this crucial role in plant defense, stomata have often been overlooked in discussions of pathogen resistance. Recent findings, however, highlight the significance of stomatal closure induced by bacterial pathogen-associated molecular patterns (PAMPs) like flagellin and lipopolysaccharide (LPS), supporting the idea that stomata play a vital role in plant innate immunity [2]. For instance, coronatine, which chemically resembles methyl jasmonate, can undo bacteria-induced stomatal closure, allowing pathogens such as *Pseudomonas syringae* pv. *tomato* to access leaves even after the initial stomatal reaction [3]. Furthermore, the formation of biofilms aids epiphytic bacterial phytopathogens, such as *Xanthomonas axonopodis* pv. *citri*, in survival and colonization, with *Xcc* occasionally entering leaves through stomata in *Brassicaceae* [4]. The rpf/DSF gene cluster controls the secretion of a chemical by *Xcc*, which regulates stomatal closure in *Arabidopsis* [5]. Studies have reported the inhibition of PAMP and ABA-induced stomatal closure in *Arabidopsis* by *Xcc* supernatants and extracts, highlighting the importance of secreted factors in pathogenicity [6]. *Arabidopsis* MPK3 has also been found to be crucial for PAMP-triggered stomatal closure, as both chitosan and yeast-derived elicitors induce plant defensive responses by elevating guard cell-free cytosolic Ca^2+^ [7].

The review highlights the importance of stomata in plants for gas exchange and its significance as an entry point for phytopathogens. While some studies have explored stomatal responses to pathogens, the overall role of stomata in plant defense has been understudied. Recent research has shown that stomata play a crucial role in plant innate immunity, with pathogen-induced closure and various signaling pathways influencing their behavior. This review aims to justify the need for a fresh examination of stomatal defense mechanisms, emphasizing unique and meaningful aspects that have not been extensively explored before, and providing a comprehensive and updated perspective on the topic, such as new signaling components and pathways involved; an in-depth analysis of the role of specific hormones, such as abscisic acid (ABA) and jasmonic acid (JA), involved in stomatal closure and defense; an examination of the potential applications of stomatal manipulation in crop protection strategies; and a critical evaluation of the current challenges and gaps in our understanding of stomatal closure and defense.

## 2. The Quest to Focus on Stomatal-Based Resistance in Plants against Pathogen

Stomatal signaling pathways and defense mechanisms are crucial for plants to combat invading pathogens. Pathogen-associated molecular patterns (PAMPs) trigger stomatal closure as a first line of defense. Hormones, such as ABA, play a role in regulating stomatal closure, and recent research has identified stomatal receptor proteins for pathogen detection. The stomatal microbiome influences stomatal-mediated resistance, and understanding genetic regulation and environmental interactions enhances plant defense. Engineering stomatal-based resistance shows promise in enhancing crop protection against pathogens (Table 1).

## 3. Natural Plant Structure Interacting with Pathogens during Invasion

Morphological and anatomical adaptations, chemical–physiological defenses, and physical attributes all work in tandem to create barriers and conditions that hinder pathogen entry, growth, and colonization. In addition, these factors play crucial roles in influencing stomata, defense mechanisms, and some other important structures, and their roles are discussed below (Table 2).

## 4. Stomatal Exclusion in Plant–Fungi Interactions 

**Figure 1:**
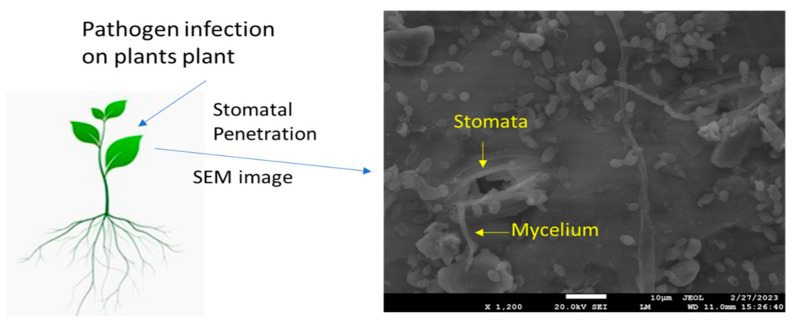
Scanning electron microscopic image shows deposition of fungal mycelium (*Bipolaris maydis*) on maize leaf upon infection.

Stomatal exclusion is a vital defense mechanism in plants, preventing pathogen entry and protecting against infections [29]. Rust fungi, such as the leaf blade-specialized rust fungi *Puccinia triticina* and *P. coronata*, causing wheat leaf rust and oat crown rust, respectively, have been suggested as the cause of the low prevalence of leaf sheath and peduncle infection in cereal crops through stomatal exclusion, also known as failed stoma penetration. The stem-specific stem rust fungus invades the stomata in leaf sheaths and peduncles at considerably higher rates than the leaf blade-specific leaf and crown rust fungi [29,30]. According to these findings, the degree of stomatal exclusion varies among cultivars, and intriguingly, the mechanism in charge appeared to extend to the leaf blade on the oat cultivar “Garry”; however, a more recent study was unable to substantiate the notion that stomatal exclusion shields leaf sheaths from *P. triticina* and *P. hordei*. In general, rust germination on leaf blades appears to be quite rare, and host genotype variations were insignificant or unpredictable [31]. Current research on maize demonstrated that the number of stomata, size of stomata, and type of cultivar all affect the penetration of fungi [32,33,34]. However, the susceptible variety exhibits a wider stomatal aperture and greater fungal mycelium deposition around the stomata (Figure 1). There are variations in the rust fungus *Uromyces viciae*-stomatal fabae’s exclusion amongst faba bean cultivars [35]. Some accessions had fewer substomatal vesicles that formed outside of the leaves than inside the substomatal cavity. These differences were substantial, but they were too small to be anticipated to have a significant epidemiological effect [36]. Minor changes in the stomatal exclusion of the barley leaf rust fungus, brought on by the formation of the substomatal vesicle prior to stoma penetration, were also discovered in a group of barley eceriferum mutants; however, once more, the frequencies were too low to affect the epidemic. In the case of diseases other than rusts, it has been observed that germ tubes may leave stomata after entering them, and it has been reported that this event occurred in tomato/*Cladosporium fulvum*, where the tomato genotype had a strong gene for resistance to hypersensitivity, but the author did not quantify this component [37]. One such study suggests that stomatal closure is induced by chitin and chitosan present in the fungal cell wall [38]. The level of stomatal exclusion may be significant in various plant pathosystems, such as powdery mildew fungi, which are another group of pathogens known to interact with stomata during their infection process. These fungi are spread by the germination of spores on the surface of plants. These spores’ germ tubes have the ability to actively enter the plant tissues via the stomata. Once within the substomatal cavity, the fungus grows and creates haustoria, which are feeding structures that draw nutrients from the host cells [39]. According to some studies, the stomata size and distribution can affect how well powdery mildew spores penetrate surfaces. Different plant species and cultivars may exhibit differences in stomatal density, size, and aperture, which may influence their susceptibility to powdery mildew infection. The efficacy of stomatal exclusion against powdery mildew fungus might also depend on host genetic variables [40].

## 5. Stomatal Response to Bacteria Invasion and Signaling Components

Stomata were previously thought to be passive points of entry, raising the possibility of bacterial attacks through open stomata; however, recent research has shown that stomata in the *Arabidopsis* plant actively respond to living bacteria, sophisticated pathogen-associated molecular patterns (PAMP), and microbe-associated molecular patterns (MAMP) [41,42]. Bacterium-induced stomatal closure requires PAMP signaling and salicylic acid (SA) homeostasis, working in tandem with ABA-controlled signaling in the guard cells. Conversely, the PAMPs and bacterial recognition in stomatal guard cells are interconnected [43]. Plants utilize LRR receptors, such as the flagellin receptor FLS2, to detect PAMPs. Both LPS and Tu are essential elongation factors, highly conserved bacterial substances that trigger induced innate immune reactions, similar to flagellin [44,45]. The flg22 PAMP was unable to seal the stomata in *Arabidopsis* fls2 flagellin receptor mutant epidermal peels. This suggests that the homologous PAMP receptor is necessary for guard-cell sensing of flg22. The production of nitric oxide is accelerated by the flagellin 22 (flg22) and LPS in wild-type stomatal guard cells within 10 min [46]. Additionally, SA and ABA were found to be necessary for the PAMP signal transduction pathway, as well as the stomatal response to bacteria or PAMPs [47]. Neither flg22 nor LPS causes stomatal closure in the ABA biosynthetic mutant [48] or the ABA signaling mutant [46,49]. The stomata of *Arabidopsis* nahG or eds16 plants having SA deficiency do not react to PAMPs either [46]. Here, SA is a potent inducer of stomata closure; hence, it is puzzling that SA-deficient plants do not exhibit the same level of PAMP/bacterium-triggered stomatal closure. This evidence suggests the molecular link between PAMP, SA, and ABA signaling in guard cells in response to bacterial invasion. There is a possibility that the ABA, SA, and PAMP signaling networks can operate concurrently in the guard cell and are connected by specific branches. The pathway of the guard cells might be changed by a fault in the SA and ABA signaling networks, indirectly affecting PAMP signaling. Furthermore, it is yet uncertain if PAMP detection by immunological receptors, such as FLS2, results in increased synthesis of SA and ABA. There may be unique signaling characteristics in stomatal guard cells that make cell type-specific studies necessary.

## 6. Hypersensitivity Reaction (HR), Stomatal Closure, and Pathogenesis

Recent research has shed light on how hypersensitivity responses may influence stomatal behavior and vice versa [50]. The hypersensitive response happens once the pathogen breaks through the plant cell wall and starts the formation of haustorium or intracellular hyphae [51]. HR is a plant defense mechanism that is characterized by the rapid death of cells at the site of infection, which creates a physical barrier that prevents the pathogen from spreading [52]. Stomatal closure and the hypersensitive response (HR) are triggered by pathogens, PAMPs, elicitors, and oxidative stress induced by hydrogen peroxide (H_2_O_2_), NO, and ROS. Oxidative stress, in conjunction with ABA, is linked to stomatal closure, and the signal transduction network activated by ABA is one of the most well-characterized signaling processes in guard cells. This suggests that HR and the stomatal response to stress are interconnected, especially in biotrophic pathogens [50]. One such study on ABA mutant *Arabidopsis* suggested that those plants that showed insensitivity towards ABA exhibited reduced stomatal opening [53]. ABA is associated with ROS generation in guard cells and ROS is associated with HR reaction in plants [54]. Several examples suggest the link between ROS, the pathogen response, and the stomata. Another study provides evidence that the expression of the *FeSOD1* gene can help to protect tomato plants from infection by *Phytophthora infestans* [55]. This suggests that stomata may play a role in the development of HR and that the *FeSOD1* gene could be a potential target for the development of new strategies to control this pathogen. 

## 7. Molecular Mechanisms of Stomatal Response to Pathogens 

The molecular response is linked with cytosolic NADPH, the substrate of the NADPH oxidases, and ROS production. There is a correlation between the ABA-induced stomatal closure and an increase in ROS and free cytosolic Ca^2+^ [56]. A study showed that antisense MPK3 plants do not respond to phytopathogens or H_2_O_2_ and exhibited normal closure promotion in response to ABA. In contrast, ABA signaling promotes stomatal closure in these cells redundantly, whereas PAMP signaling in the cells is completely dependent on H_2_O_2_, necessitating the presence of MPK3. ABA is known to activate a range of signaling events in guard cells [16]. Pathogen-induced indirect inhibition of H^+^ ATPase activity mediated by H_2_O_2_ is demonstrated by research on the *Xcc* factor. This hypothesis proposes that guard cells express *Arabidopsis* RIN4, a negative regulator of plant immunity [57]. These plants are hypersensitive to coronatine because H^+^-ATPase activity and pathogenic *Pst* are unable to open rin4 mutant stomata. The fusicoccin toxin also inhibits H^+^-ATPase, but it does so through a different method that necessitates direct protein interaction [58]. In a cell density-dependent manner, the cell-to-cell communication pathway rpf/DSF controls biofilm development and fungal endophyte colonization-associated gene expression, which includes xanthans, plant defense suppression, and glucans [59]. Further, the rpf/DSF gene cluster plays a crucial role in regulating fungal endophytes Xcc colonization through various mechanisms. This gene cluster is associated with a decrease in plant innate immunity and alters stomatal responses. Interestingly, it appears that biofilm formation may not be necessary for bacterial stomatal penetration, even though it facilitates endophytic colonization. This process can occur in isolated epidermis with or without biofilm formation when coronatine or the *Xcc* factor is present [7]. Furthermore, unlike the Xcc factor, the fungal toxin fusicoccin significantly increases stomatal opening, indicating a different mechanism at play. Research has revealed that stomatal behavior is influenced by the phytopathogenic fungus *Plasmopara viticola* and *Rhynchosporium secalis*, as well as virulence factors or fungal metabolites, such as oxalic acid, that also promote stomatal opening [60,61]. The intriguing potential that stomatal innate defense-overriding mechanisms are more widespread than previously believed and that they independently developed in various pathogens is raised by the aforementioned cases. Intriguing new tools for studying stomatal physiology may come from the discovery of additional pathogen compounds involved in modulating stomatal defense as well as their targets inside guard cells. These findings may also lead to the identification of novel strategies to prevent pathogen penetration into the leaves. 

## 8. ABA Response to Biotic and Abiotic Stresses during Stomatal Regulation

ABA levels rise when plants are subjected to water stress. Insufficient soil moisture may be interpreted by roots as a cue to start ABA synthesis from starch [62]. When ABA is increased in the foliar part, this is also connected with drought-related ABA in the roots, implying that drought-induced ABA substantially alters the water potential of leaves [63,64]. To promote stomatal closure and reduce water loss through transpiration, ABA transports from the roots to the leaves [65]. In these circumstances, water loss and pathogen ingress can be reduced by increasing ABA and closing the stomata [66]. A study suggests that plants infected with the pathogen *Colletotrichum* show an increased level of ABA [67]. Another study suggests that ABA levels during infection were related to clonal variability in chestnuts during susceptibility or resistance to *Phytophthora cinnamomi* [68]. It is difficult to determine the precise link between endogenous ABA levels and susceptibility to disease in plants because it depends on the length of the infection, additional stressors, and pathotype [69]. ABA has been reported to show resistance during the early stages of pathogen infection [70]. More research is needed to properly comprehend the diverse impacts of ABA on pathogen sensitivity modulation, particularly in relation to plant tissue predisposition. A finding suggests that in plants, ABA activates cyclic nucleotide-gated channels (CNGC) in guard cells [71]. This activation initiates ABA-specific calcium signaling, crucial for stomatal closure in *Arabidopsis*. The CNGC channels in the plasma membrane of guard cells allow the influx of calcium ions, regulating the stomatal opening and closing process. This mechanism enables plants to respond to environmental cues and conserve water during periods of stress, ultimately aiding in their survival and adaptation. On the other hand, during drought stress, SPR1 positively regulates microtubule disassembly in ABA-induced stomatal closure. This process relies on OST1-mediated phosphorylation, highlighting the connection between ABA signaling and MAPs in regulating plant responses to drought [72,73].

## 9. Pattern-Triggered Immunity (PTI) and Pathogen–Stomatal Interaction 

Most microbiological pathogens can generate pathogens or microbes (PAMPs, MAMPs), and pattern recognition receptors (PRRs) recognize these signals on the plasma membrane of the plant. When plants recognize stress, they launch a defense mechanism, PTI (pattern-triggered immunity) [74]. Bacterial pathogens release elicitor peptides, such as elf26, LPS, and flagellin22 (flg22), to cause stomatal closure [46]. On the other hand, there are many fungal elicitors, such as chitin oligosaccharide and chitosan, responsible for inducing plant defense responses [38]. Pathogen resistance is provided by elevated levels of ROS, nitrogen oxide (NO), calcium ions (Ca^2+^), and hydrogen sulfide (H_2_S) [75,76]. *Arabidopsis thaliana*’s OSCA1.3 calcium-permeable channel controls stomatal closure during immune signaling. It undergoes rapid phosphorylation upon detecting pathogen-associated molecular patterns (PAMPs). The immune receptor-associated kinase BIK1 interacts with and phosphorylates OSCA1.3’s N-terminal cytosolic loop within minutes of exposure to the PAMP flg22. This study reveals the channel’s activation mechanisms during immune signaling, indicating specificity in calcium influx responses to various stresses [77].

## 10. Stomatal Closure: An Immediate Microbial Entry Barrier and Primary Response to an Array of Stress Condition

The primary stress response is closure, which provides inherent resistance to infections [78]. Physical barriers, which are present on the outside of the plant, such as the epidermis, and the cell wall may shield it from biotic and abiotic impacts. On the other hand, various germs can easily enter through the tiny openings known as stomata that are found on leaf surfaces. These apertures in the leaves permit microbial entry, photosynthetic gas exchange, and transpiration. In terms of sensing and reacting, stomatal guard cells are extremely sensitive to foreign microbial infections. A major defensive tactic against abiotic and biotic hazards, including drought and diseases, is stomatal closure [79]. Elicitors or other chemical compounds cause stomatal closure where the leaves produce salicylic acid (SA), methyl jasmonate (MJ), etc. in response to pathogens (Table 3). Stomata are able to recognize and react to molecular patterns (MAMP) of the bacteria, including chitosan, flagellin, and harpin. To detect ABA or other substances and trigger stomatal closure, a similar signaling pathway, including receptors, protein kinases, secondary messengers, ion channels, ion efflux, and turgor loss in guard cells, is involved. *OST1* is a key NADPH oxidase activator among kinases that increase ROS levels in the guard cells. During ABA-induced stomatal closure, an increase in *OST1* kinase is usually followed by activation of *RBOH*, resulting in ROS and Ca^2+^ levels. Ca^2+^-dependent protein kinases (*CPKs*) are activated in the K^+^ out channels, the S-type anion channel 3 (*SLAH3*), and the outflow of ions from guard cells increases, forcing the stomata to close. When a yeast elicitor of microorganisms or flg22 is present, the activity of *OST1* does not change or increase [80]. Even though it was in a dormant condition, *OST1* participated in stomatal closure in response to a variety of signals, such as PAMPs, or environmental factors, such as high carbon dioxide (CO_2_) or high humidity. In addition to its activity via ROS/NO/Ca^2+^ events involving *OST1/SnRK2s*, *OST1* was found to directly control ion channels in order to cause stomatal closure in current studies [81]; however, some studies also claim that biotic and abiotic stresses can cause stomatal closure that is “*OST1*-independent” [82,83]. Plant elicitor peptides, a group of molecular patterns linked to damage, induce stomatal closure by activating *SLAC1* and *SLAH3* without *OST1* [83]. Similarly, elevated CO_2_ bypasses the *OST1* kinase to activate SLAC1. For instance, signaling activities in guard cells can activate SLAC1/SLAH3 through the MAPK cascade. The precise mechanism is uncertain; however, MAPK 3/6 was implicated in stomatal closure in the dark, and MPK 9/12 activated SLAC1 by integrating with the Ca^2+^/*CPKs* [84]. Additional leaf elements, such as trichomes, callose or silicon deposition, cuticular waxes, and callose and silicon deposition, can also resist biotic and environmental stresses [85,86,87]. 

## 11. Stomatal Closure Mediated by Hormones and Ions during Stress

Numerous additional compounds rise when plants are under stress, such as ABA, which closes the stomata and supports the plant’s defense response. The two types of substances that fit into this category are secondary metabolites and hormones (Table 3). A complicated web of signaling processes transduces the ABA signal in guard cells, generating mixtures such as nitric oxide and H_2_O_2_, cytosolic Ca^2+^ fluctuations, the guard cell tangible OST1 kinase, and other signaling intermediates. Ion channel regulation is the result of the signaling processes that ABA finally initiates, such as the GORK1 potassium channel in the guard cell, which controls the guard cells’ ion outflow. The release of ions by guard cells drives water flow and affects the turgor of the guard cells, causing the stomatal pores to close. MJ is the most effective, which encourages stomatal closure by elevating pH, ROS, NO, and Ca^2+^-like ABA activates anion channels [92]. More research is needed to completely understand how ET and BRs affect closure. A plant defense hormone called SA also has elicitor functions [97]. Reactive oxygen species, which are mostly produced by peroxidase, are involved in the SA-induced stomatal closure, unlike NADPH oxidase in ABA. 

NO production in guard cells is required for ABA-induced stomatal closure [98]. As a result, SA and ABA-regulated signaling pathways in Arabidopsis overlap to trigger stomatal closure. The interaction between ROS and NO may provide resistance to pathogens; on the other hand, considerable amounts of proline (osmolyte) may bring only partial closure [99]. Polyamine oxidase raised the amounts of ROS and NO after oxidizing polyamines (PAs), which triggered stomatal closure similar to that brought on by ABA [16]. Several other compounds support the defense mechanism of stomatal closure in response to different stresses. Stress tolerance is increased by ABA’s interaction with the aforementioned hormones, elicitors, and metabolites (Table 3). ABA is well known for interacting with MJ or SA to induce stomatal closure and pathogen resistance. As research has shown, MJ boosted ABA synthesis in *Arabidopsis* by activating AtNCED3 gene expression [92]; when SA acts on the stomata, ABA is necessary [100]. Increased ABA, on the other hand, promotes stomatal closure by activating SID2 and initiating SA biosynthesis. Mitogen-activated protein kinases (MPK9 and MPK12) regulate the signaling of ABA during stomatal closure induced by SA [101]. These two kinases are also known for regulating chitosan-induced stomatal closure [102]. One study showed that ABA-mediated NO production is dependent on H_2_O_2_ generation for stomatal closure [103]. Uncertainty surrounds the function of PAs and proline in the host tissue’s pathogen resistance, which facilitates infection transmission. ABA can still aid the plant’s defense, even under virus infection. ABA is known to activate certain behaviors involving the hypersensitive response (HR) and long-term adaptation, either on its own or in concert with other hormones such as SA or MJ to ensure enhanced resistance (Table 4).

## 12. Relation among Ethylene, ABA, and Stomatal Closure

The role of ethylene in stomatal closure is complex and depends on a number of factors. Ethylene can have both inhibitory and stimulatory effects on stomatal closure. It can inhibit ABA-induced stomatal closure by accumulating flavonols, which repress ABA-induced ROS production and stomatal closure. It can also stimulate NADPH oxidase AtRbohF-dependent H_2_O_2_ production through the activation of the Gα protein in Arabidopsis guard cells, leading to stomatal closure. A mathematical model suggests that an increase in either ethylene or ABA alone results in stomatal closure, whereas the presence of both hormones diminishes stomatal closure [109,110]. On the other hand, ethylene is also responsible for the defense response during host–pathogen interaction [111,112]. A study found that ethylene is involved in the sugarcane–smut interaction [113]. The study used a cDNA-AFLP analysis to identify genes that were differentially expressed in sugarcane plants after infection with the smut fungus. It was found that a group of genes related to the ethylene pathway were differentially expressed in sugarcane plants after infection. This suggests that ethylene is produced in response to the infection and that it is involved in the regulation of genes that are involved in defense. This suggests that ethylene plays a role in stimulating the production of these defense proteins, which can help to protect the plant from the fungus. One possible explanation is that ethylene may cause the stomata to close, which would help to protect the plant from the fungus by reducing the amount of oxygen and water vapor that is available to the fungus.

## 13. Signaling Mechanism in the Guard Cell during Pathogen Invasion 

**Figure 2:**
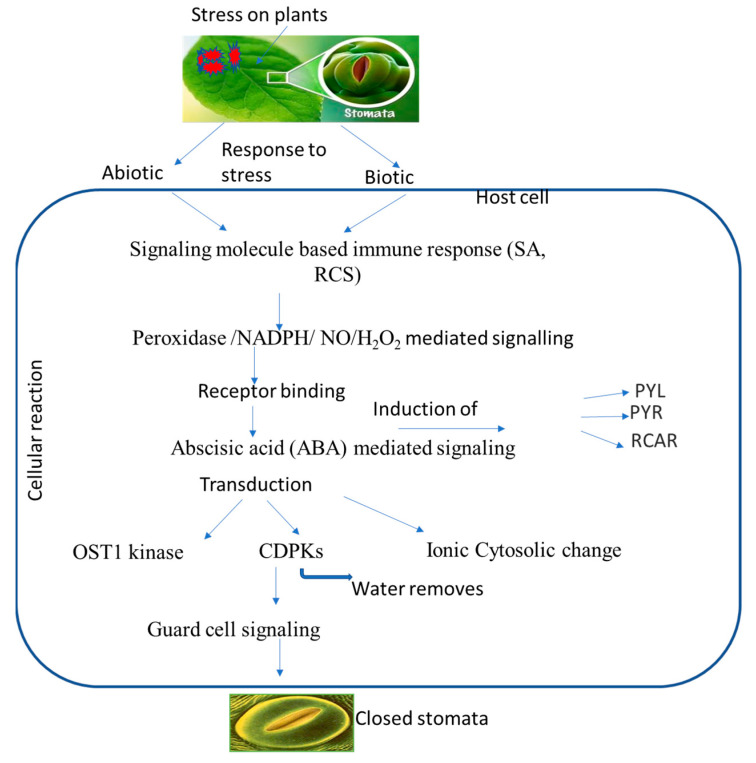
Putative overview of stomatal-mediated defense against plant pathogens at cellular level induced by ABA. Biotic and abiotic stresses induce signaling molecules, and ion generation takes place, which subsequently induces ABA. ABA binds to receptors, which leads to a change in the equilibrium of *OST1* kinase and calcium ions that ultimately leads to stomatal closure.

Increased ionic efflux causes a decrease in guard cell turgor pressure, which plays a role in stomatal closure. A defined transduction pathway is responsible for the events wherein ABA or another chemical triggers stomatal closure, and previous studies have demonstrated the importance of ABA in stomatal regulation. ABA binding to its receptor leads to the inactivation of protein phosphatase 2C, resulting in the activation of OST1 kinase. This, in turn, triggers NADPH oxidase to produce ROS and then NO. Both ROS and NO can increase cytosolic Ca^2+^ levels. High concentrations of ROS, NO, and Ca^2+^, either alone or in combination, activate anion/cation efflux channels and inhibit inflow channels. As a consequence, the loss of cations and anions from guard cells leads to turgor loss and subsequent stomatal closure [16]. Three secondary messengers, namely, ROS, NO, and Ca^2+^ can trigger the production of other signaling molecules, such as phospholipase, phosphatidic acid, and inositol 1,4,5-triphosphate. These molecules, in turn, lead to an increase in pH and subsequent stomatal closure. On the other hand, well-known NO and other gas transmitters, including CO and H_2_S, are also connected to the ABA-induced stomatal closure. Reactive carbonyl species (RCS), a different signaling element, have just recently been found to be crucial for stomatal shutdown. RCS are a group of *α*,*β*-unsaturated carbonyl compounds produced from lipid peroxides which play an important role in stress-elicited stomatal closure during ABA activity [114]. They are also known as damage mediators of ROS downstream during programmed cell death (PCD), root injury, stomatal response to ABA, etc. All of these investigations suggest that RCS and ABA may help guard cells respond to both abiotic and biotic stress. Several signaling components are activated during ABA-induced stomatal closure, which can protect cells from pathogens (Table 3). ABA creates primary and secondary messengers, such as ROS, NO, and Ca^2+^, which possibly start defensive reactions, including stomatal closure and PCD [115,116]. As a signaling molecule, ABA-induced NO can cause adaptive reactions to biotic (pathogens or elicitors) and abiotic (UV, drought, or salt) factors. It was discovered that the reaction products of ROS and NO and NO-mediated post-translational modifications can contribute to initiating defense responses [117,118]. Elevated cytosolic Ca^2+^ was typically required to trigger HR as a plant immunological response, for instance, to microbial pathogens. The ability of plants to fight off infections has also been related to other ABA signaling molecules; phospholipase D and phosphatidic acid are two examples [119]. Stomatal closure and plant pathogen adaptation may be connected, as evidenced by the ability of the gas transmitter H_2_S to provide resistance against the common bacterial disease *Pseudomonas syringe* [120]. The formation of ROS and NO, which might be a key component in plant defense, can be encouraged or inhibited by gas transmitters. The plant species *Arabidopsis thaliana* has been shown to be a useful model for investigating and confirming the mechanisms and constituents of plant function, and *A. thaliana* mutants were employed to identify the signaling elements of ABA (Table 5). Three types of these mutants are distinguished: those with altered ABA production or reception, those lacking signaling molecules, and those whose stomatal response is unaffected by ABA. Mutants with open stomata lose their defenses against infections and develop a heightened sensitivity to them. These results demonstrate the close relationship between increased pathogen resistance and ABA or chemically related substances that cause stomatal closure (Figure 2).

## 14. Stomata in Competitive Interactions with Plant Pathogens and Herbivores

Herbivore-induced stomatal closure has some effects on the competition connections between insects and microbes. Several diseases occur due to the entry of pathogens into plant cells through stomata [46]. Some herbivorous insects introduce nonpathogenic microorganisms, which results in an increase in the JA defense response and further triggers the SA defense response in plants [85]. Infection by a pathogen can cause herbivores to lose important plant resources; as such, insect herbivores may have developed as a response to protect plants from various diseases. Salivary glucose oxidase (GOX), which has recently been proven to cause stomatal closure, is produced by a large number of insect herbivores [128]. There is a conservatory component present in insects, i.e., GOX, which produces H_2_O_2_ [129]. Likewise, many microbes produce GOX, which holds off microbial competition. To prevent plant pathogen infection, caterpillar salivary GOX may inhibit the growth of the pathogen by producing H_2_O_2_, which promotes stomatal shutdown. Sucking insects, such as aphids, induce SA defense reactions in plants, such as microbial breaching, as opposed to feeding on herbivores. As a result, insect-feeding guilds and microbial species are expected to play a role in the benefit of limiting microbial invasion. SA and JA defenses have been reported to be combative, and some herbivores may occasionally retain stomatal openings to ease pathogen contagions, even though there is evidence that stomatal closure plays a role in mediating herbivore–microorganism interactions [130]. Stomatal closure may be a part of plant anti-herbivore defense signaling and is regarded to be the primary mechanism of the herbivore-persuaded photosynthetic hindrance. Even though photosynthesis produces essential molecules for the creation of chemicals associated with defense, blocking growth and photosynthesis typically leads to an increase in defense. Long recognized as the result of resource reallocation, trade-offs between growth and defense now appear to be significantly influenced by JA-associated signaling networks. According to new research, the defensive signaling network most likely includes the ability to detect and decrease carbon assimilation. It was discovered that tobacco plants respond more defensively to higher levels of leaf damage, and it was hypothesized that plants detect vandalism by determining the degree of carbon source restriction. Additional studies are required to discover if some of the damaged signs that modify the growth–defense equilibrium include herbivore-persuaded stomatal shutdown, which indecently influences carbon absorption. There is an increase in photosynthesis action per unit leaf area after an attack by chewing insect herbivores, in contrast to fast depletion in the stomatal hole and photosynthesis in the herbivory [131]. Much research has been conducted to determine whether plant forbearance and recuperation from herbivory are connected to enhanced photosynthetic activity. This is most likely due to an increase in the desire for photosynthesis in sink tissues because of herbivory’s direct or indirect limitation of carbon absorption. Increased photosynthesis following herbivory may also be a quiet plant response to increased systemic nutrition availability, such as nitrogen, that restricts photosynthesis and upgrades the water status following tissue mislaying; it opens the stomata and promotes greater photosynthetic activity [132]. The ability of plants to tolerate defoliation is associated with overexpressed photosynthesis, it could be an active process to counteract the fitness costs of herbivory. Stomata may be involved in controlling a range of herbivory responses because of the strong connections between stomatal dynamics and photosynthesis defense; however, the physiological mechanisms connecting these activities have remained a mystery.

## 15. Stomata Manipulation by Insects

There is mounting evidence that insect herbivores alter stomata. The stomata and guard cells are crucial defense mechanisms against invading pathogens, such as *Bipolaris maydis* and insects, where insects might alter the stomatal dynamics on which they feed and allow invading pathogens to enter [133]. Many biocontrol agents have the capacity to suppress the pest population in the host plant [134,135,136]. Some investigations have found that plants’ experiences of herbivory or modification by herbivores, as opposed to artificial harm, result in altered stomatal responses. Interactions between tobacco hornworm larvae and winter moth-pedunculate oak larvae are two examples. *Pieris brassicae* and *Spodoptera* larvae oral secretions reduce wound-induced leaf water loss relative to mechanical damage alone, indicating increased stomatal closure [131]. Eating by leaf miners or moth larvae has been shown to boost the efficiency of water consumption by 200 percent when feeding on whole leaves. Mined leaves with larvae performed better than mined leaves without larvae, showing higher stomatal closure and suggesting active stomatal manipulation. These results suggest that herbivore-associated molecular patterns (HAMPs) are responsible for stomatal closure, although the particular HAMPs and physiological processes involved in the closure are unknown. The salivary GOX of *Helicoverpa zea* larvae that increases stomatal closure in tomato and soybean plants also plays a crucial function in H_2_O_2_-producing GOX in herbivore-induced plant volatiles (HIPVs) suppression and is one strong mechanism that conducts stomatal closure provoked by HAMPs, while the conductivity of cotton stomata was reported to be unaltered by GOX in the same study. Stomatal closure is brought on by specialized herbivores, which reduces the number of HIPVs that draw in natural enemies; however, there is little proof that specialists can close stomata, and it is unclear what this means for the environment. In addition to the possible restriction of HIPVs, stomatal closure is already associated with a decrease in secondary metabolite translocation, such as nicotine, which is created in tobacco (*Nicotiana tabacum*) roots and transferred to the leaves. In *Helicoverpa zea* larvae and *Manduca sexta* larvae, salivary GOX and oral secretions have been observed to decrease the stomatal aperture and nicotine concentration in plant leaves. There are not many possible explanations for this relationship.

## 16. Regulation of Temperature and Water Availability upon Herbivory Attack

Stomata closure may aid insect herbivores by increasing the temperature and moisture content of plant tissues. When leaves are damaged, the rate of transpiration from the wounds frequently increases. Inducing stomatal closure may help herbivores maintain leaf water content after injury. Aphids and other piercing–sucking insects can promote stomatal closure, which further lowers transpiration and preserves leaf water potential in addition to herbivores who consume leaf tissues. These modifications lead to longer feeding periods and an increase in aphid abundance. Stomatal closure affects the microenvironment of the leaf as well. Stomata closing increases leaf warmth and decreases transpiration [137]. Studies show that plant stomatal closure enhances aphid feeding, which may directly help herbivores by hastening their growth [138] and shortening vulnerable life stages [139], thus lowering the danger of being preyed upon by size-limited carnivores while reducing predator–prey geographical overlap [140].

## 17. Conclusions

Research on host-pathogen resistance in stomata reveals a crucial front in host–pathogen interactions. Stomata, tiny pores on the surface of leaves, allow plants to exchange gases with their surroundings. Stomatal guard cells are extremely susceptible to external microbial infections and can recognize and respond to the molecular patterns associated with bacteria. Plants have acquired the ability to modify their stomatal apertures in response to pathogens and environmental conditions, and these apertures serve as effective entry points for phytopathogens to colonize endophytes. ABA triggers stomatal closure in guard cells, which is accompanied by an increase in ROS and free cytosolic Ca^2+^. Although it is known that PAMPs and bacteria promote stomatal closure, the mechanisms by which stomatal guard cells detect these chemicals are still developing. Both the PAMP signal transduction pathway and the stomatal response to bacteria require SA and ABA, and their concentrations must reach at least the minimum level for PAMP signaling in the guard cell. Stomatal closure, contributing to water status maintenance and providing innate infection resistance, represents one of a plant’s earliest responses to stress. When plants are exposed to water stress or insect attacks or are under attack from pathogens, polyamine oxidase builds up and takes part in the defense mechanism. Another important aspect is that the stomatal closure carried by ABA raises the ROS and NO levels. During ABA-induced stomatal closure, a few signaling components are triggered that can protect cells from pathogens, including ROS, NO, and Ca^2+^. On the other hand, stomatal closure increases the warmth and water content of plant tissues, which is advantageous to insect herbivores. The discovery of the host–pathogenic interaction at the stomatal level thus represents a significant conceptual advance in the understanding of pathogenesis, stomatal biology, the microbial ecology of plants, various types of biotic and abiotic stress, interactions between herbivores, and various types of insect manipulation, as well as changes in the phyllosphere temperature, which can then be used in resistant breeding programs or to develop a climate-resilient variety.

## Figures and Tables

**Table 1:** Recent signaling components and pathways involved in stomatal closure and defense.

Signaling Components and Pathways	Description	References
Calcium signaling	Involves novel calcium-permeable channels and sensors in guard cells, mediating stomatal closure.	[8]
Reactive oxygen species (ROS) signaling	Specific ROS species act as secondary messengers, regulating ion channels and enzymes for stomatal closure.	[9,10]
Small peptides and hormones	Newly discovered stomagen and SCF peptides modulate stomatal movement during pathogen attacks.	[11]
Protein kinases and phosphatases	Recently identified kinases and phosphatases regulate stomatal closure and downstream events.	[12]
G-protein coupled receptors (GPCRs)	Novel GPCRs serve as receptors for pathogen molecules, activating downstream stomatal responses.	[13]
RNA-based regulation	Small RNAs (microRNAs, lncRNAs) fine-tune stomatal responses through post-transcriptional control.	[14]
Epigenetic regulation	DNA methylation and histone modifications influence gene expression in stomatal defense.	[15]
Crosstalk between phytohormones	Interactions between ABA, JA, and SA coordinate stomatal responses during pathogen challenges.	[16]
Nutrient sensing pathways	The TOR signaling pathway has been linked to stomatal closure, suggesting nutrient influence.	[17]
Post-translational modifications	Ubiquitylation, phosphorylation, and SUMOylation regulate key proteins involved in stomatal closure and plant immunity.	[18]

**Table 2:** Critical factors shaping plant defense strategies against pathogens: morphological, anatomical, chemical, and physical influences.

	Morphological and Anatomical Features		
Natural Structure	Function	Host-Pathogen Example	References
Raised Stomata	Positioned on the upper surface of leaves, these stomata might offer some protection against direct pathogen contact due to their elevated position.	Beet-*Cercospora beticola*	[19,20]
Submerged Stomata	Common in aquatic plants, submerged stomata might face fewer pathogens due to the water layer that acts as a barrier.	Wheat-*Puccinia striiformis f.* sp. *tritici*	[21,22]
Specialized Stomata	Found in desert plants, these stomata could be adapted to minimize water loss, potentially impacting the invasion of waterborne pathogens.	Citrus-*Xanthomonas citri* subsp. *citri*	[23]
	**Chemical–Physiological Defenses**		
Epidermal Waxes	These waxes create a physical barrier that prevents pathogens from directly reaching plant cells, reducing the risk of invasion.	Barley-*Blumeria graminis*	[24]
Exudates	Chemical compounds released from waxes can hinder pathogen growth. These compounds might have antimicrobial properties that directly deter pathogens.	Tomato-powdery mildew-*Oidium neolycopersici*	[25,26]
**Physical Characteristics**			
Physical Characteristics	Moist cell walls in the aerenchyma support gas exchange and overall plant health. Cuticles provide structural integration. This environment might be less favorable for certain pathogens, reducing their ability to colonize and invade.	Rice-*Magnaporthe oryzae*	[27]
Internal Cavity Water Vapor	Water vapor within the internal cavity maintains humidity levels, creating conditions that support defense mechanisms. It might also affect pathogen survival by influencing moisture-dependent processes.	*Pseudomonas syringae* pv. *tomato* in tomato	[28]

**Table 3:** Molecules associated with regulation of stomatal response on the stomata closure.

Molecule	Reaction to a Pathogen	References
ABA	Induces stomatal closure during pathogen invasion, e.g., *Leptosphaeria maculans* and *Pseudomonas syringae*.	[88,89]
Ethylene	To enhance resistance against *Magnaporthe oryzae,* production *of* ROS and phytoalexin.	[90]
Chitin and Chitosan	Chitin and chitosan not only independently but together also capable of inducing stomatal immunity against fungal pathogen.	[38]
Allyl isothiocyanate (AITC) and methyl jasmonate	Induces stomatal closure leading decreases in water loss and pathogen invasion as reported in *Arabidopsis* plant.	[91,92]
Cryptogein and harpin	Elicitors of tobacco pathogen were capable of causing stomatal closure.	[93]
Cerato-platanin	Induces hormone signaling, which triggers PAMP leads to reduction in fungal infection.	[94]
Cyclodipeptides	ROS, cytosolic Ca^2+^, and NO production for stomatal closure; activation of *PR-1a* gene and protein and increment in cellular SA levels for reducing *Phytophthora nicotianae* and *Tobacco mosaic virus* infections in tobacco.	[95]
Cytokinin	In response to *Agrobacterium tumefaciens*, an HR-like response, cell death, and PR gene activation were all induced.	[96]

**Table 4:** Examples of various compounds and their interactions responsible for stomatal closure.

Compound	The Impact on the Stomata	Name of the Plant	References
β-aminobutyric acid (BABA)	Drought causes ABA to accumulate.	*Triticum aestivum*	[104]
Salicylic acid (SA)	Well-established messenger and inducer of disease resistance, endogenously or exogenously.	Wide range of crops for local and systemic pathogen	[97]
γ-Aminobutyric acid (GABA)	Reduces the invasion of anions into the vacuole and represses 14-3-3 proteins	*Arabidopsis thaliana*	[105]
Lipopolysaccharide (LPS)	Nitric oxide synthase (NOS) is activated and NO is produced in guard cells.	*Arabidopsis thaliana*	[106]
Methyl Jasmonate (MJ)	H_2_O_2_ production and cytoplasmic alkalinization are aided by this compound.	*Arabidopsis thaliana*	[107]
Oligogalacturonic acid (OGA)	Expands the amounts of cytosolic Ca^2+^ and ROS.	*Lycopersicon esculentum*; *Commelina communis*	[108]
Harpin	Elicitor	*Arabidopsis thaliana*	[93]

**Table 5:** Examples of *Arabidopsis* mutants for ABA and their response to pathogen.

Altered Plant Compounds and ABA Production	Pathogen-Induced Responses	References
*aba2-12* and *aao3-2* hamper ABA biosynthesis	*Pythium irregulare* susceptible	[121]
*aba3-1* is essential for the biosynthesis of ABA	flg22 and LPS failed to seal the wound	[41]
ABA insensitive (*abi1*, *abi2*)	Did not close the stomata in response to *Trichoderma* species	[122]
Subunits of G-proteins (Gα, Gβ and Gγ)	Open stomata extremely vulnerable to *Pseudomonas syringe pathogens*	[123]
MAPKs (*mpk3*, *mpk6*)	In response to PAMP or Pst, not effective	[124]
Open stomata 1 (*ost1*) reduces K^+^ efflux	Flg22 induced rapid stomatal closure	[125]
Enhanced response to ABA1 (*era1*) is associated with the farnesyl transferase subunit	ABA hypersensitivity as well as pathogenic microbes	[126]
Lipoxygenase (*lox1*)	The ability of stomata to seal in response to bacteria and LPS is impaired	[127]

## Data Availability

Not applicable.
